# Polystyrene Thin Films Nanostructuring by UV Femtosecond Laser Beam: From One Spot to Large Surface

**DOI:** 10.3390/nano11051060

**Published:** 2021-04-21

**Authors:** Olga Shavdina, Hervé Rabat, Marylène Vayer, Agnès Petit, Christophe Sinturel, Nadjib Semmar

**Affiliations:** 1 GREMI (Groupe de Recherches sur l’Energétique des Milieux Ionisés)-UMR (Unité Mixte de Recherche) 7344-CNRS, University of Orleans, 45067 Orléans, France; herve.rabat@univ-orleans.fr (H.R.); agnes.petit@univ-orleans.fr (A.P.); nadjib.semmar@univ-orleans.fr (N.S.); 2 ICMN (Interfaces, Confinement, Matériaux et Nanostructures)-UMR (Unité Mixte de Recherche) 7374-CNRS, Université d’Orleans, 45071 Orléans, France; marylene.vayer@cnrs-orleans.fr (M.V.); christophe.sinturel@univ-orleans.fr (C.S.)

**Keywords:** polymer thin films, femtosecond beam, laser texturing, LSFL, 2D LIPSS, substrate temperature effect, scanning processing

## Abstract

In this work, direct irradiation by a Ti:Sapphire (100 fs) femtosecond laser beam at third harmonic (266 nm), with a moderate repetition rate (50 and 1000 Hz), was used to create regular periodic nanostructures upon polystyrene (PS) thin films. Typical Low Spatial Frequency LIPSSs (LSFLs) were obtained for 50 Hz, as well as for 1 kHz, in cases of one spot zone, and also using a line scanning irradiation. Laser beam fluence, repetition rate, number of pulses (or irradiation time), and scan velocity were optimized to lead to the formation of various periodic nanostructures. It was found that the surface morphology of PS strongly depends on the accumulation of a high number of pulses (10^3^ to 10^7^ pulses) at low energy (1 to 20 µJ/pulse). Additionally, heating the substrate from room temperature up to 97 °C during the laser irradiation modified the ripples’ morphology, particularly their amplitude enhancement from 12 nm (RT) to 20 nm. Scanning electron microscopy and atomic force microscopy were used to image the morphological features of the surface structures. Laser-beam scanning at a chosen speed allowed for the generation of well-resolved ripples on the polymer film and homogeneity over a large area.

## 1. Introduction

Periodic structured surfaces at micro- or nanometric scales find applications in a wide variety of fields, such as microelectronics [1], optics [2], photovoltaics [3], tribology [4], biological sensors [5], and anti-counterfeiting [6]. The generation of periodic surface structures can be performed by using different methods, for instance, nanoimprint [7], which is limited by the necessity to manufacture the micro- or nanostructured molds. The lithographic method requires clean-room facilities and multi-step procedure involving complex mask fabrication [8]. Laser patterning can be a powerful technique for high-resolution surface nano-engineering [9,10,11,12]. It is a non-contact, mask-less, and flexible/versatile method with direct generation of micro- and nano-structures called laser-induced periodic surface structures (LIPSSs) observed on a wide range of materials, such as semiconductors [13,14], metals and alloys [15,16,17,18,19], metal oxides [20,21,22,23], inorganic compounds [24,25], and polymers [26]. Micro- and nano-structures could also be simultaneously observed in hierarchical patterns [19,24].

The formation mechanisms of LIPSS are still under investigation and have been described by several theories, including interference between the incident and scattered waves [27], self-organization [25], second-harmonic generation [28], and excitation of surface plasmon polaritons [29]. Redistribution of the energy through a feedback mechanism enhances surface structuring [30]. The dimensions of the structures, as well as their morphology, depend on the laser-beam texturing parameters. The periods of the generated structures are strongly correlated to the wavelength of the laser source [31], and the linear polarization allows us to form regular ripple structures parallel to the polarization [32].

Some polymers were structured in the bulk state by femtosecond laser, namely poly(methyl methacrylate) PMMA [33], polystyrene PS [34], polydimethylsiloxane PDMS [35], polytetrafluoroethylene PTFE [36], and polyether ether ketone PEEK [37]. Potential applications of structured polymer surfaces are nano-templating, storage media applications [3], and some specific solutions for biosystems [38]. By direct irradiation of PTFE plate [36], micro–nano dual-scale composite structures were created. The micro-grooves morphology of LIPSS was formed thanks to Ti:Sapphire laser on PMMA microchip [33]. Due to the particular properties of the polymers, such as low glass transition temperature (T_g_), the LIPSS formations on polymer foils are often achieved with low beam fluencies (with a fluence of 10.5 and 12.5 mJ/cm^2^ for PET and PS irradiated by nanosecond laser beam at 248 nm) [26].

Only a few polymer thin films, such as polyethylene terephthalate PET, polytrimethylene terephthalate PTT, polyvinylidene fluoride PVDF, and polycarbonate bisphenol A PC, were irradiated by nanosecond laser in order to create periodic structures with a period (Λ) similar to the laser wavelength (Λ~λ) and parallel to the laser polarization direction [39]. Moreover, several studies on LIPSS generation in polymer films were conducted by using solid-state laser-beam sources from the fundamental wavelength, typically 1064 nm with Nd:YAG sources down to the fourth harmonic at 266 nm [32], and in other works, with excimer sources from 193 to 308 nm [40].

Considering the laser regime in the literature, femtosecond beams are less employed for polymer-thin-films processing [41,42], and particularly with UV beams, as investigated in the present work. The advantage of the femtosecond (fs) regime compared to the nanosecond one is that the LIPSS could be formed despite the low absorption coefficient of the polymers [43]. In the fs regime, non-linear absorption and ionization processes mediate the coupling of laser light with the thin films, as reported in Reference [44].

In this work, we first studied femtosecond multi-pulse laser irradiation at 266 nm on spin-coated PS thin films within a single spot zone. More specifically, using low energy values (few µJ/pulse), we studied the influence of laser frequency (50 and 1000 Hz) and number of laser pulses (from 5 × 10^3^ to 800 × 10^3^ pulses) on the surface morphology and on the LIPSS main features (period and amplitude). The influence of the substrate temperature on LIPSS morphology, rarely explored to our knowledge, was also investigated thanks to a controlled heating Peltier module.

Using a XY-translation stage, we created ripples over large area (few mm length and width) and optimized the different parameters for this process. The surface morphology of the irradiated films was systematically analyzed by scanning electron microscopy (SEM) and atomic force microscopy (AFM).

## 2. Materials and Methods

**Figure 1:**
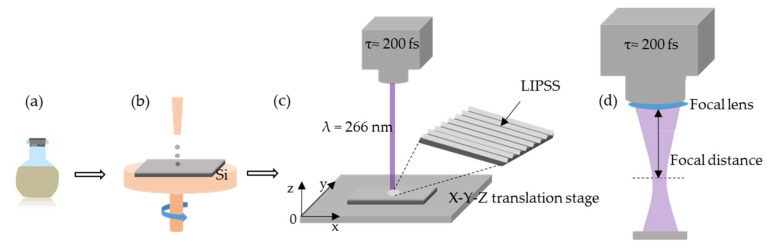
Schematics of PS laser surface structuring steps: (**a**) preparation of polymer solution by dissolution of the polymer in solvent, (**b**) spin-coating of polymer solution onto silicon substrate to obtain a supported polymer film, (**c**) irradiation of polymer film by fs laser, and (**d**) laser-beam focus using a focal lens.

The schematic of the procedure leading to LIPSS formation on polymer films is shown in Figure 1. Firstly, polymer solution was prepared by dissolution of polystyrene in dichloroethane (30 mg/mL) (PS with a molecular weight of 170 kg·mol^−1^ was purchased from Sigma-Aldrich (Merck KGaA, Saint-Louis, MO, USA)). Silicon wafers were cut into pieces of 1.5 × 1.5 cm^2^. These substrates were cleaned with water, ethanol and acetone. Spin-coating of polymer solution was carried out at the speed of 4000 rpm, during 30 s, on silicon substrates (Figure 1b). The deposited polymer film’s thickness (around 180 nm) was determined by using a DektakXT Stylus profilometer (Bruker Corporation, Billerica, MA, USA).

PS-thin-film irradiations were carried out with femtosecond laser beam at a wavelength of 266 nm, thanks to the third harmonic generator of a Ti:Sapphire source (800 nm, 100 fs). An estimation of the temporal duration of these UV pulses is about 200 fs, as reported in Reference [45]. The Spectra Physics Mai Tai laser system (Mai Tai Diode-Pumped, Mode-Locked Ti:Sapphire Laser, Spectra-Physics Inc., Mountain View, CA, USA) used in our experiment consists of two parts: a CW diode-pumped Nd:YVO_4_ laser and a mode-locked Ti:Sapphire pulsed laser. We used linearly polarized laser pulses with a Gaussian beam spatial profile. The beam spot size and, thus, the fluence were adjusted by the distance between the focal lens and the sample XY-translation stage, at normal incidence parallel to the Z-axis (Figure 1d). The working distance (from focusing lens to substrate surface) was determined to be close to 78.5 mm in the single spot case. The control of the pulse number (N) for 50 Hz and 1 kHz repetition rates was achieved thanks to an electronic shutter. The experiments were performed under standard laboratory conditions, ambient atmosphere, and room temperature (RT).

To study the effect of substrate temperature on the structure morphology, the substrate was heated during the irradiation, from RT to 40, 60, and 97 °C. Then the Peltier module (RS 490-1525, Adaptive, Russia) was used. In the scanning mode, only the 1 kHz repetition rate condition was applied. The same focal lens as in single spot processing was used to focalize the laser beam. The velocity of the XY-translation stage displacement was between 20 and 100 μm/s. The effective number of pulses, N_eff_, was directly derived from the overlap of the spots and depends on the spot size and the scanning velocity. The working distance was determined to be close to 68.5 mm in the scanning mode, leading to the best process parameters in terms of spot size, laser fluence, and process time.

An Atomic Force Microscope (AFM) Dimension ICON from Bruker with ScanAsyst in Air mode (Bruker Corporation, Billerica, MA, USA) was used to study the surface morphologies and to compare sample roughness before and after irradiation. The morphology of the thin film was also observed after irradiation, using a Zeiss SUPRA 40 Scanning Electron Microscope (SEM) (German Carl Zeiss Company, Oberkochen, Germany).

## 3. Results

### 3.1. Single Spot Processing

**Figure 2:**
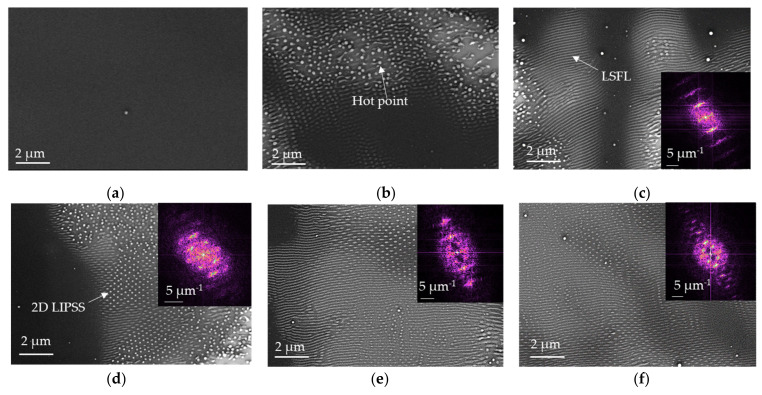
SEM micrographs of PS film with corresponding FFT images from AFM analysis: (**a**) before and after laser irradiation at pulse fluence F = 11.8 mJ/cm^2^, repetition rate of 50 Hz for pulse number N: (**b**) 5 × 10^3^, (**c**) 10 × 10^3^, (**d**) 15 × 10^3^, (**e**) 25 × 10^3^, and (**f**) 35 × 10^3^.

Figure 2 shows SEM micrographs of PS film before (Figure 2a) and after laser irradiation (Figure 2b–f) for 50 Hz of repetition rate, 8 µJ of pulse energy, and 11.8 mJ/cm^2^ of fluence per pulse. The generation of LIPSS structures in PS requires a minimum number of pulses to start the modification of the surface morphology. Within our experimental parameters, the first structures started to emerge from 5 × 10^3^ pulses (Figure 2b). These structures are probably formed around microscopic defects generation (hot points) and film roughness, as previously suggested [33]. These few-micrometers hot points grow and merge into larger areas. At N corresponding to 10 × 10^3^ pulses, the micrographs reveal the formation of 1D periodic parallel structures (Figure 2c) that can be attributed to a low spatial-repetition rate LIPSS (LSFL). During the subsequent pulses, probably through the radiation–structuration feedback mechanisms, LSFL structures emerged as dominant structures. By further increasing the number of shots up to 15 × 10^3^ shots, we observed nanodots structures with a 2D periodicity (2D-LIPSS) from Figure 2d. For the number of pulses between 15 × 10^3^ and 35 × 10^3^, LSFL and 2D-LIPSS were observed simultaneously within the same irradiation spot. From AFM analyses for the same samples characterized by SEM, the 2D Fast Fourier Transformation (FFT) was used in order to analyze the periodicities of LIPSS (Figure 2). From FFT analysis, we found that, for 10 × 10^3^ pulses, LSFL has a period of Λ~165 nm, while above 15 × 10^3^ pulses, the observed 2D-LIPSS exhibit a 2D periodicity of Λ_1_~165 nm and Λ_2_~230 nm. For 25 × 10^3^ and 35 × 10^3^ pulses (Figure 2e,f), from 2D-FFT analysis, the same 2D orientation and periodicity are observed.

From classical electromagnetic theory, it is known that the period, Λ, of the ripples depends on the laser wavelength, λ, effective refractive index, n, and the angle of incidence of the radiation, θ:

(1) $$\Lambda =\frac{\lambda }{n\pm \sin\left(\theta \right)}$$

Thanks to the ellipsometric measurement, we found a refractive index of 1.57 for PS film at 266 nm, using Cauchy model. PS film was irradiated without tilting, and the calculated period of LIPSS corresponds to Λ~170 nm, confirming theoretical approach. This type of structure is induced by interference between incident wave and scattered and reflected waves that creates a modulation of the intensity of the beam in thin film.

**Figure 3:**
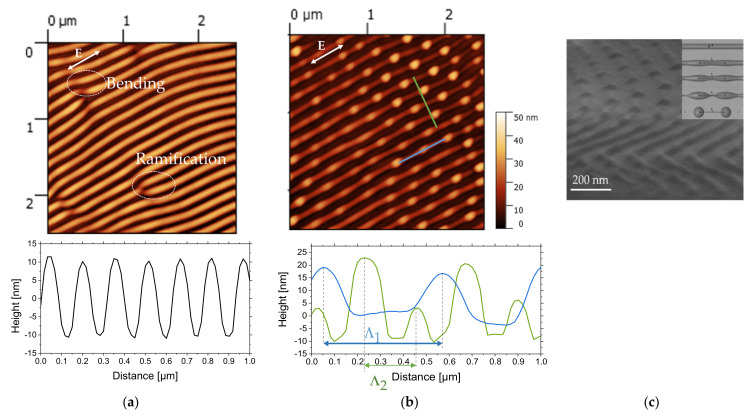
AFM height (amplitude) images and cross-section profiles along the corresponding lines (2.5 × 2.5 µm^2^ size, Z scale is 50 nm) of PS film irradiated at 266 nm, at a repetition rate of 50 Hz, a fluence of 11.8 mJ/cm^2^, after 35 × 10^3^ pulses: (**a**) area 1, (**b**) area 2, and (**c**) SEM image. The polarization direction of the laser beam is indicated by double arrows.

Figure 3a,b presents AFM images of PS film after 35 × 10^3^ pulses of laser irradiation and the corresponding cross-section profiles. Figure 3c displays a SEM micrograph of the tilted sample exhibiting ripples in the foreground and 2D-LIPSS in the background.

From AFM images on area 1 (Figure 3a), ripples with a typical height of 20 nm can be observed with some bendings and ramifications. The ramifications are characterized by the separation of the ripples into two or three separated ripples. These types of structures are associated with a spatial modulation of absorbed energy by defects on polystyrene thin film and superposition of the LIPSS wave vectors. The same types of structures were observed on silicon surface [46] and were explained by combining two-temperature model, free-carrier dynamics and Sipe’s theory [47].

From Figure 3b, another type of structure is observed on area 2, characterized by the formation of 2D-LIPSS within the ripples. From the AFM image, 2 periodicities can be observed with Λ_1_~340 nm and Λ_2_~165 nm. 2D LIPSSs with a typical height of 30 nm are formed inside parallel LSFL structures with a typical height of 13 nm. We suggest that formation of these nanodots is dominated by hydrodynamic instabilities, resulting in material redistribution from parallel ripples. We suppose that local instabilities are induced by surface-tension minimization that results in the ripple breaking into smaller parts. The viscosity of polymers is decreasing during irradiation, and hydrodynamic phenomenon starts to play a dominant role. The presence of two types of structures, namely ripples and nanodots, is the result of viscosity variations and a combination of broken and unbroken LSFL. Hexagonal lattice types have also been studied by M. Fermigier et al. [48], where the nonlinear growth of the Rayleigh–Taylor instability leads to the formation of two-dimensional patterns with hexagonal shape.

**Figure 4:**
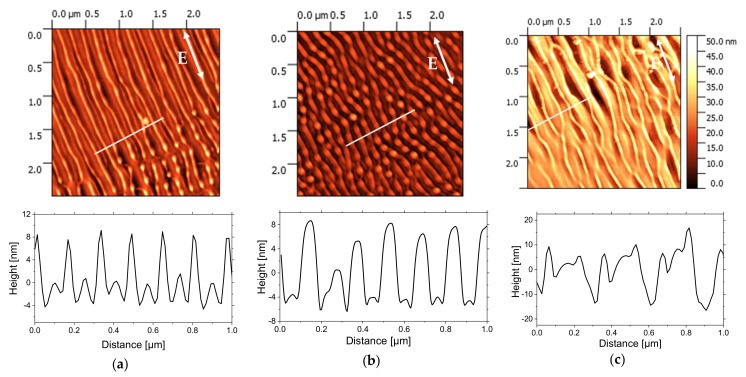
AFM height images (2.5 × 2.5 µm^2^ size) and cross-sections profiles along the corresponding white lines of PS films irradiated at 266 nm, 1 kHz of repetition rate, and at F = 0.6 m${J/\mathrm{cm}}^{2}$, with number of pulses, N, (**a**) 50 × 10^3^, (**b**) 100 × 10^3^, and (**c**) 800 × 10^3^. The polarization direction of the laser beam is indicated by double arrows.

In order to produce the periodic structures at higher laser repetition rate and reduce the duration between pulses down to 1 ms, the irradiations were also carried out with the repetition rate of 1 kHz. Figure 4 displays AFM height images of 2.5 × 2.5 µm^2^ area of PS films irradiated with 50 × 10^3^, 100 × 10^3^, and 800 × 10^3^ pulses for 0.6 mJ/cm^2^ of pulse fluence. For the lowest number of pulses (Figure 4a), first morphological changes are observed with the creation of periodic parallel structures. The period is Λ~170 nm, and the height of the structures is up to 10 nm. With the increasing of the pulses number up to 100 × 10^3^, nanodots start to appear with the decreasing of the period Λ down to 150 nm. The height of these nanodots is around 10 nm. Finally, for an even higher number of pulses corresponding to 800 × 10^3^ (Figure 4c), the parallel structures start to be distorted with the irradiated area exhibiting an array of like-groove morphologies. The distance between two consecutive grooves is around Λ~250 nm, and their depth is around 25 nm.

It was proposed by He and al. [46] that the quasi-periodic pattern of stripes on silicon is related to a spatial redistribution of the absorbed energy and to the formation of the regions where the absorbed fluence is not enough to induce effective ablation and could fuse the surface nanostructures leading to the progressive generation of the groove stripes covering the underlying ripples. We suppose that the same phenomenon was observed for PS film and reactions of photolysis are generated and induced the material loss. These photochemical reactions are characterized by much shorter time scales than thermal-related processes.

Based on Sipe’s theory [47], wave vectors are possible from LIPSS, depending on surface parameters (surface roughness and dielectric permittivity) and laser irradiation parameters. Once the LIPSSs are formed, surface roughness will induce the further enhancement of the ripples’ formation characterized by the redistribution of optical energy from subsequent laser pulses. In other words, we find the feedback effect, which explains the increase on the surface of the ripples. It is important to underline that the ripple formation is extended over a large area, and this is related to repeat scattering during multi-pulse irradiation, which can propagate the pattern.

Irradiation at 50 Hz can be compared with irradiation at 1 kHz, using the accumulated dose, which is the fluence per pulse times the number of pulses. The accumulated dose (60 J/cm^2^) at 50 Hz for 5 × 10^3^ pulses is the same as the one at 1 kHz for 100 × 10^3^ pulses, and the accumulated dose (413 J/cm^2^) at 50 Hz for 35 × 10^3^ pulses is similar as the one (480 J/cm^2^) at 1 kHz for 800 × 10^3^ pulses.

### 3.2. Influence of the Substrate Temperature on the LIPSS Morphology

**Figure 5:**
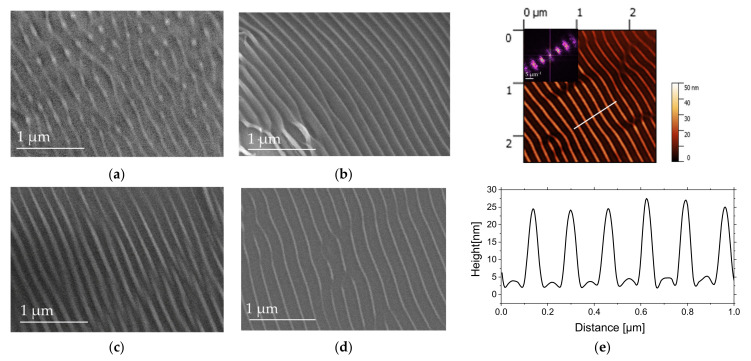
SEM images of PS film irradiated at λ = 266 nm,1 kHz of repetition rate and pulse fluence F = 0.6 m${J/\mathrm{cm}}^{2}$ with 10^5^ pulses at (**a**) 23.5 °C, (**b**) 40 °C, (**c**) 60 °C, and (**d**) 97 °C. (**e**) AFM height image (2.5 × 2.5 µm^2^ size) of PS film irradiated at 266 nm, 1 kHz of repetition rate with number of pulses N = 100 × 10^3^ at 97 °C, and cross-section profile along the white line.

It is known that the behavior of polymers strongly depends on the temperature [49]. From a microscopic point of view, their behavior is associated with molecular relaxations and the elastic modulus decreases above T_g_ (here measured for PS bulk by DSC at 95 °C, in accordance with the value estimated by Vignaud et al. [50] at 97 °C for a PS 135 nm–thick layer). To investigate the influence of the substrate temperature, the ripples were generated with number of pulses N = 10^5^, which is optimal for the formation of organized structures at a fixed fs-laser pulse fluence (F = 0.6 m${J/\mathrm{cm}}^{2})$ and laser repetition rate (1 kHz). This allowed us to create ripples parallel to the polarization vector. We varied the temperature of the Peltier module from RT to 97 °C, heating the polymer film during the irradiation. Figure 5 displays the corresponding SEM images. LSFLs are observed with the same period between ripples Λ~170 nm whatever the temperature of the substrate. However, the width of the ripples decreases from 80 nm at RT (Figure 5a) to 55 nm for 97 °C (Figure 5d). From AFM images, it could be seen that the height of the ripples was increased from 10 nm for RT (Figure 4b) to 20 nm for 97 °C (Figure 5e). The LIPSSs are formed independently of PS thickness, indicating that structuration process is significant only in the surface layer, which absorbs the light of the laser beam.

At low temperature corresponding to 23.5 °C, chains of the polymer are in a glassy state. Increasing of the temperature causes an increase in the mobility of macromolecular chains within thin film. The application of the laser energy at a temperature higher than RT can promote the passage of PS in soften state (Figure 5b) [51]. The softening of the PS makes hydrodynamic contributions, including thermo-capillary flow to the LSFL mechanisms, dominant, leading to higher and narrower ripples.

### 3.3. Scanning Processing

**Figure 6:**
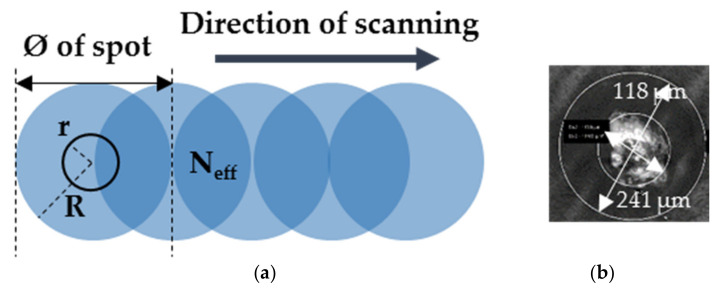
(**a**) Schematic of the beam overlap during a scanning process. (**b**) SEM image of laser impact on PS film irradiated at 266 nm at the repetition rate of 1 kHz at z = 68.5 mm.

We also proceed to the LIPSS generation in scanning mode in order to create periodic structures on large area. In order to optimize the scanning time, we approached the substrate towards the FoF position to z = 68.5 mm from the focusing laser lens. Figure 6a illustrates the beam overlap during the scanning process. There are two zones in the laser spot to be consider, as shown in Figure 6b: The first one in the middle of the laser spot, with a diameter equal to 2r, is the zone where there are many photons interacting with the PS layer and where soft ablation can take place in relation to the Gaussian beam shape. There is also the weak zone of interaction of the laser beam with the layer, with the outer diameter 2R.

For this, the scanning was carried out at different speeds, ν, for a repetition rate, ω, corresponding to 1 kHz, which then defines a number of effectives pulses, N_eff_, that applied during the overlap of two laser spots with their diameters, Ø, corresponding to 241 µm:

(2) $$N_{\mathrm{eff}}=Ø\frac{\omega }{\nu }$$

The average number of laser pulses in overlapping area was thus ranged from 2410 to 12,050 by varying the scanning speed of the laser beam from 20 to 100 μm/s at fixed fluence corresponding to 2.2 mJ/cm^2^.

**Figure 7:**
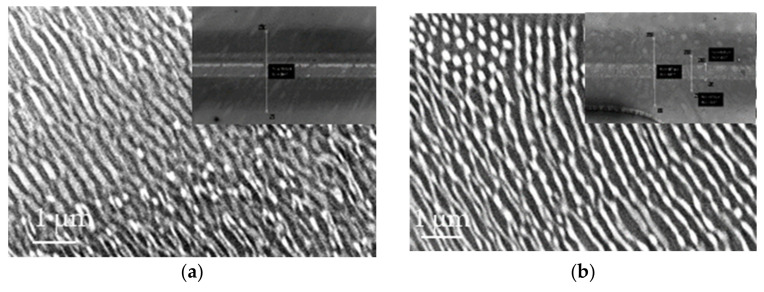

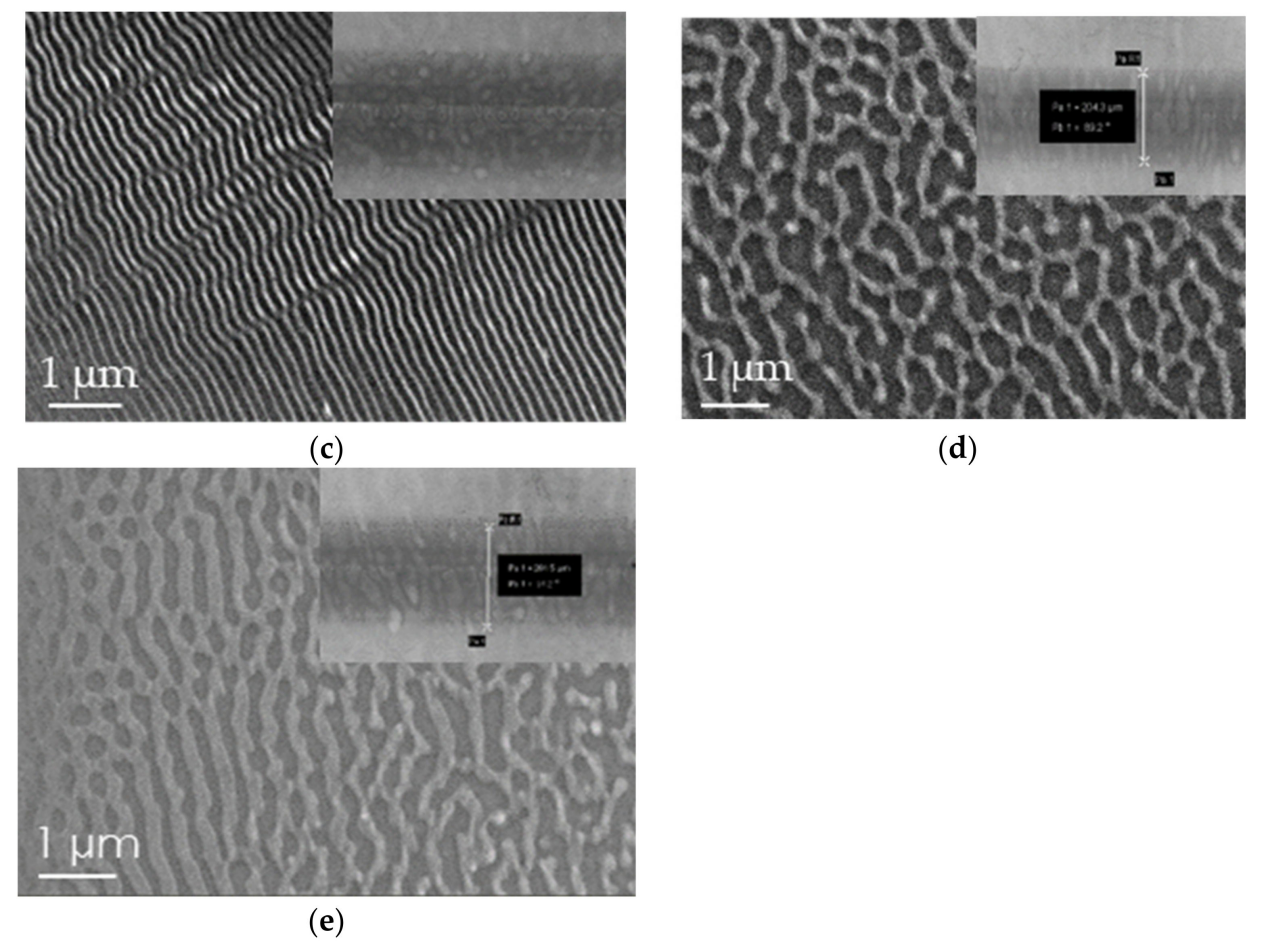
SEM images of PS film irradiated at λ = 266 nm at frequency of 1 kHz and pulse fluence of 2.2 m${J/\mathrm{cm}}^{2}$ at different scanning speeds: (**a**) 20, (**b**) 40, (**c**) 60, (**d**) 80, and (**e**) 100 μm/s.

Figure 7 presents SEM images of the formed structures. At the microscopic scale, the periodic parallel structures for 20 µm/s are created with Λ~170 nm (Figure 7a). At low scanning speed, a soft ablative regime dominates in the center of the spot with the LIPSS generation around this area. By increasing the speed until 40 µm/s, the polymer is less affected in central part of laser impact (Figure 7b). For a scanning speed of 60 μm/s, the quasi-parallel ripples are dominant and cover a large area of scanning line (Figure 7c). Higher speeds (80 and 100 µm/s) lead to the same result, with observed quasi-periodic structures with higher period (Figure 7d. This is related to the fact that the effective number of pulses of the laser beam in the overlap zone is not sufficient to induce the LIPSS.

**Figure 8:**
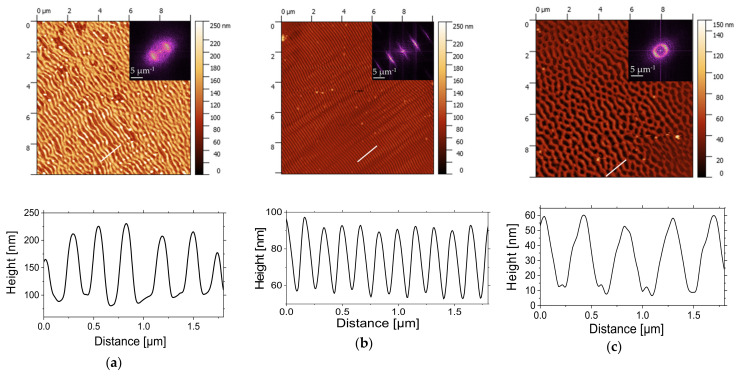
AFM height images (10 × 10 µm^2^ size) and corresponding 2D-FFT scattering graphs of PS film irradiated at λ of 266 nm with repetition rate of 1 kHz and a pulse fluence of 2.2 m${J/\mathrm{cm}}^{2},$ at scanning speeds (**a**) 20, (**b**) 60, and (**c**) 80 µm/s.

From AFM images (Figure 8a–c) and corresponding 2D-FFT image analyses, it can be seen that, at a lower scanning speed corresponding to 20 µm/s (with N_eff_ = 12,050), the structures are linearly oriented with height H~130 nm but partially destructed. This could relate to the material loss due to the photochemical reactions initiated by the absorption of energy of laser beam with the creation of transient excited states and future consequence of bond breaking and radicalization. For a scanning speed of 60 µm/s, well-defined and periodic LSFLs are observed. The height of ripples is about 35 nm, which could be related to the material redistribution with modulated energy deposition but not material loss. When radiation order couples with the surface structure order, one enhances the other, and periodic structures grow faster than disordered ones and emerge as a dominant surface feature. The generation of higher-order harmonics from 2D-FFT analysis highlights the good quality and homogeneity of the ripples on large areas for this scanning speed with the period of Λ~170 nm and the height of H~35 nm. A scanning speed of 80 µm/s leads mostly to an elliptic profile of 2D-FFT, with a higher period corresponding to 400 nm.

**Figure 9:**
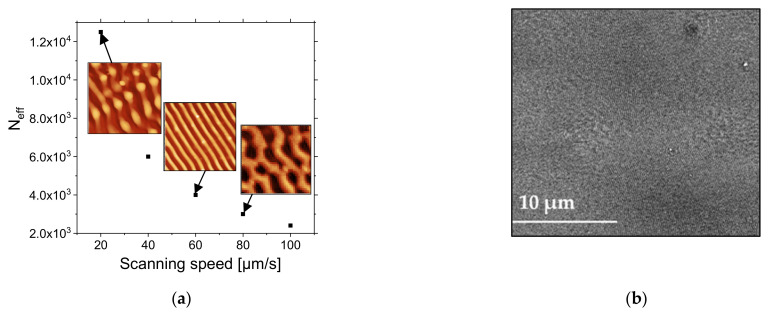
(**a**) Average effective number N_eff_ of pulses at overlapping area as a function of scanning speed (from 20 to 100 µm/s). (**b**) SEM image of PS film irradiated at λ 266 nm, with a repetition rate of 1 kHz and a pulse fluence of 2.2 m${J/\mathrm{cm}}^{2}$ at a 60 µm/s scanning speed.

As shown in Figure 9a, the effective number of pulses decreases from 2410 to 12,050 when the scanning speed increases from 20 to 100 µm/s. The conditions for well-defined and organized LIPSS were optimal for an effective number of pulses of 4000 for a pulse fluence of 2.2 mJ/cm^2^. These conditions allowed us to obtain LIPSS without any defect, except for bending over a large area, as shown in Figure 9b. The size of the area is limited by the size of the scanning area. The test was performed over 4 × 4 mm^2^.

## 4. Conclusions

We performed PS thin films nanostructuring by UV femtosecond laser beam at 266 nm, with two repetitions rates corresponding to 50 and 1000 Hz. For 50 Hz of repetition rate and 11.8 mJ/cm^2^ of fluence per pulse, parallel LSFL structures were observed above 10^3^ pulses structures with a period Λ smaller than the laser irradiation wavelength λ. Ramifications and bendings of ripples were also observed, and they are probably associated with the spatial modulation of the absorbed energy by PS-thin-film defects on polystyrene thin film. Two-dimensional LIPSS structures started to appear for pulse numbers greater than 15 × 10^3^. For 1 kHz of repetition rate, 0.6 mJ/cm^2^ of fluence per pulse and a number of pulses between 50 × 10^3^ and 800 × 10^3^, we found parallel structures LSFL with the period close to 170 nm and less than the beam wavelength. For 800 × 10^3^ pulses, the ripples started to be destroyed with the formation of the groove-like morphology with a spatial period of 260 nm. Scanning the PS film with the laser beam also generated LIPSS on large areas, and we found that 60 µm/s was the optimal speed for the creation of well-resolved ripples for a 2.2 mJ/cm^2^ of fluence per pulse.

Finally, we demonstrated that heating the PS films from RT up to 97 °C upon femtosecond laser irradiation simultaneously induced the ripples’ width decrease and amplitude increase.

## Data Availability

The data is included in the main text.
